# Comorbidity genetic risk and pathways impact SARS-CoV-2 infection outcomes

**DOI:** 10.1038/s41598-023-36900-z

**Published:** 2023-06-19

**Authors:** Rachel K. Jaros, Tayaza Fadason, David Cameron-Smith, Evgeniia Golovina, Justin M. O’Sullivan

**Affiliations:** 1 The Liggins Institute, The University of Auckland, Auckland, 1023 New Zealand; 2 Maurice Wilkins Centre for Molecular Biodiscovery, The University of Auckland, Auckland, 1010 New Zealand; 3 College of Health, Medicine and Wellbeing, The University of Newcastle, Callaghan, 2308 Australia; 4 MRC Lifecourse Epidemiology Unit, University of Southampton, Southampton, UK; 5 Singapore Institute for Clinical Sciences, Agency for Science, Technology and Research (A*STAR), Singapore, Singapore; 6 Australian Parkinson’s Mission, Garvan Institute of Medical Research, Sydney, NSW Australia

**Keywords:** Viral infection, Protein-protein interaction networks, Computational biology and bioinformatics, Gene regulation, Genetic association study

## Abstract

Understanding the genetic risk and mechanisms through which SARS-CoV-2 infection outcomes and comorbidities interact to impact acute and long-term sequelae is essential if we are to reduce the ongoing health burdens of the COVID-19 pandemic. Here we use a de novo protein diffusion network analysis coupled with tissue-specific gene regulatory networks, to examine putative mechanisms for associations between SARS-CoV-2 infection outcomes and comorbidities. Our approach identifies a shared genetic aetiology and molecular mechanisms for known and previously unknown comorbidities of SARS-CoV-2 infection outcomes. Additionally, genomic variants, genes and biological pathways that provide putative causal mechanisms connecting inherited risk factors for SARS-CoV-2 infection and coronary artery disease and Parkinson’s disease are identified for the first time. Our findings provide an in depth understanding of genetic impacts on traits that collectively alter an individual’s predisposition to acute and post-acute SARS-CoV-2 infection outcomes. The existence of complex inter-relationships between the comorbidities we identify raises the possibility of a much greater post-acute burden arising from SARS-CoV-2 infection if this genetic predisposition is realised.

## Introduction

Genome-wide association studies (GWAS) have identified genetic associations with severe acute respiratory syndrome coronavirus 2 (SARS-CoV-2) infection^1–7^, consistent with a complex genetic contribution to infection susceptibility and severity. Additionally, epidemiological studies have connected the outcome of SARS-CoV-2 infection with comorbidities including diabetes, obesity, active cancer, hypertension, and coronary artery disease^8^, all of which intensify SARS-CoV-2 health burdens^9–13^. Yet, the interactions between the genetic contributions associated with these complex comorbidities and the risk variants associated with SAR-CoV-2 infection outcomes remain unexplored. The reason for this is not that we are unaware of the need to treat SARS-CoV-2 infections holistically. Rather, characterising the potential causal mechanisms underlying the total genetic burden for SARS-CoV-2 infection outcomes and comorbidities requires an integrative translational approach that moves beyond cross-cohort genome-wide associations for single conditions. Thus, the problem lies in how we undertake studies to characterise the total genetic burden for SARS-CoV-2 infection, including the full suite of comorbid conditions, to gain a functional understanding of the mechanisms. Yet, the significant acute and long-term sequelae associated with ongoing SARS-CoV-2 infections mean that it is essential we address the interaction with comorbid conditions. Only then will we achieve a step-change in our ability to predict, treat and mitigate the worst outcomes of SARS-CoV-2 infection.

The COVID-19 Host Genetics Initiative (COVID-19 HGI) (https://www.covid19hg.org/) undertook a meta-analysis of 49,562 cases and 2 million controls across 46 distinct studies from 19 countries to identify the host genetic determinants of SARS-CoV-2 infection and the severity of the resulting disease^4^. The COVID-19 HGI identified variants associated with: (1) severe cases and (2) cases of moderate or severe SARS-CoV-2 (herein: hospitalised). Severe cases required respiratory support in hospital or died due to SARS-CoV-2; hospitalised cases were hospitalised as a result of SARS-CoV-2^4^. Mendelian Randomisation analyses, performed using 38 a priori selected phenotypes^4^, identified BMI (hospitalisation and reported infection), smoking initiation (hospitalisation), red blood cell count and height (reported infection), and Parkinson’s disease (hospitalisation European only without UKBiobank) as being causally related to SARS-CoV-2. In addition, eight genetic traits (diabetes, BMI, lupus, ischemic stroke, ADHD, coronary artery disease [CAD], smoking initiation, cigarettes per day) were genetically correlated with severity and hospitalisation. Notably, CAD was inconsistently associated with infection severity^4^, despite epidemiological studies having confirmed a strong incidence of cardiovascular disease that increased with the care setting during acute infection (e.g. infected, hospitalised, or intensive care^9^). The biological mechanisms that account for the causal and genetic relationships between SARS-CoV-2 and these conditions remain obscure.

Disease biology^14^, transcriptome-wide association study analysis^5^ and phenome-wide association studies^15–18^ have identified lung tissue and function as central for understanding the genetic risk contributed by SARS-CoV-2 associated variants. Yet, translating genetic knowledge into functional understandings of individual and shared disease processes is complicated by the fact that: (1) individual genetic variants associated with complex polygenic disorders typically have small effect sizes; (2) regulatory mechanisms are generally cell/tissue type-specific^19,20^; and (3) the functional outcomes of intergenic trait associated genetic variants are frequently associated with genes that are non-adjacent within the linear DNA sequence^21,22^. The application of regulatory genomics approaches has emerged as a promising strategy to identify GWAS variants that are enriched in regulatory regions relevant to the pathophysiological basis of a given trait^23,24^. In addition, protein–protein interaction networks and pathway-based approaches have identified ‘pathways’ where genes converge between diseases^25,26^. However, the integration of these information sources remains a complex undertaking.

Phenome-wide association studies^16^ have been used to screen SARS-CoV-2 associated risk variants for associations with known diseases or traits. These studies have identified an association between SARS-CoV-2, chromosome 3p21.31 and traits in monocytes, eosinophils, and neutrophils^17^. Similarly, the SARS-CoV-2 associated variant rs657152 (*ABO*) has been linked to 40 associations, including heart failure (OR, 1.09; 95% CI 1.03–1.14; q = 0.046) and diabetes (OR, 1.05; 95% CI 1.02–1.07; q = 0.004)^18^. Papadopoulou, et al. ^27^ identified increased risk for phlebitis and thrombophlebitis (OR = 1.11, *p* = 5.36 × 10^–8^) in severe SARS-CoV-2 cohorts and increased risk for leg blood clots (OR = 1.1, *p* = 1.66 × 10^–16^) in SARS-CoV-2 susceptible patients. Finally, 17q21.31 has previously been associated with SARS-CoV-2, red blood cells (count and distribution width), haemoglobin (levels and concentration), lung function traits and chronic obstructive pulmonary disorder (COPD)^15^. Despite these insights, the challenges associated with interpreting genetic variants identified by GWAS also applies to phenome-wide association studies insofar that functional information and tissue/cell type regulatory mechanisms are rarely addressed.

The combined genetic risks of SARS-CoV-2 comorbidities and predispositions have not been systematically investigated. Here, we assessed the function of SARS-CoV-2 variants in the lung, blood, brain and coronary artery by integrating chromatin conformation data (i.e. tissue-specific Hi-C) with common genetic variation (i.e. minor allele frequency ≥ 0.05, which designates the frequency cut-off at which the second most common allele occurs in a given population) and gene expression data (GTEx^28^) to identify spatially constrained expression quantitative trait loci (i.e. eQTLs). eQTLs are SNPs that explain variation in expression levels of mRNAs. We then performed an unbiased, de novo protein diffusion network analysis coupled with tissue-specific gene regulatory networks to identify spatially constrained eQTLs that regulate the encoding proteins, the traits, and biological pathways that link inherited risk factors for SARS-CoV-2 with recognised and unrecognised phenotypes.

## Results

### Lung protein interaction network analysis identifies known and unknown comorbidities of SARS-CoV-2 infection

**Figure 1:**
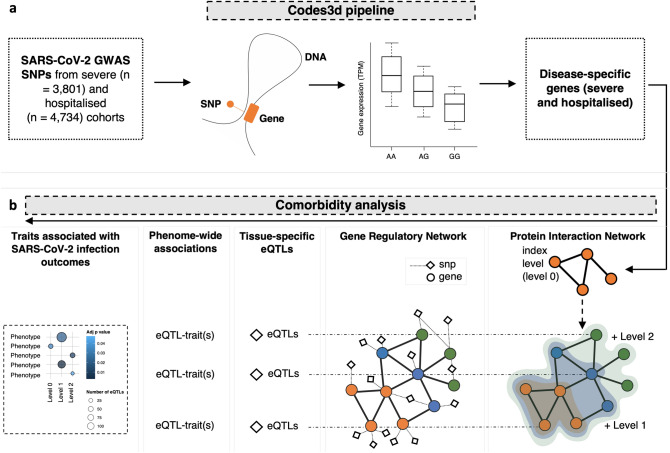
SARS-CoV-2 associated GWAS SNPs were assessed to ascertain loci functionality and identify putative mechanisms for comorbid and genetic predispositions for traits associated with SARS-CoV-2. (**a**) The Codes3D pipeline generates the index level set of target genes (Supplementary Fig. 1d) associated with the severe and hospitalised phenotypes. SNPs obtained from COVID-19 HGI^47^ (Supplementary Table 1) were screened through tissue-specific Hi-C datasets^30,53–55^ to identify *cis* (< 1 Mb), *trans* (> 1 Mb) and trans-interchromosomal SNP-gene interactions. eQTL effects were identified by testing the SNP-gene pairs against the GTEx database (version 8)^28^. The resulting statistically significant (FDR ≤ 0.05) SARS-CoV-2-specific genes (spatial eQTL-gene pairs), from both the hospitalised and severe phenotypes were assessed for protein–protein interactions using the STRING^56^ and PROPER-Seq^32^ databases. All genes were assessed using g:Profiler^57^ to obtain gene ontology terms. (**b**) The protein–protein interaction network analysis pipeline has two parts: (1) all interacting protein partners from level 0 (CoDeS3D identified index level set of genes) to level 4 were identified by querying the STRING database with parameters of high confidence threshold score > 0.7; and (2) tissue-specific gene regulatory maps were queried to obtain all known eQTLs for each protein within the expanded network. The eQTLs were then tested for enrichment (hypergeometric test) within the GWAS Catalog (https://www.ebi.ac.uk/gwas/) to identify associated phenotypes.

Proteins that interact within networks are more likely to contribute to a specific cellular process^29^. Therefore, we undertook a de novo protein interaction network analysis to explore comorbidities and predispositions associated with SARS-CoV-2 (Fig. 1). The protein interaction network was generated in two stages. Firstly, we used CoDeS3D^29^ to integrate empirically defined information on the 3-dimensional organisation of the genome in lung cells (captured by Hi-C^30^) with functional data (lung tissue expression Quantitative Trait Loci^28^ [eQTL]) to assign functional (gene expression) impacts for SARS-CoV-2 risk variants (associated with severe and hospitalised phenotypes) in lung tissue (Fig. 1a). There was a significant variant overlap between the hospitalised (71.3%) and severe (87.9%) phenotypes (Supplementary Fig. 1a). Secondly, we generated protein interaction networks by parsing the proteins encoded by the SARS-CoV-2-associated spatial eQTL targeted genes through the STRING^31^ or PROPER-Seq databases to identify proteins they directly interact with (Fig. 1b). The gene targets identified by CoDeS3D (Supplementary Fig. 1) formed level 0 (index set, n = 227; Supplementary Table 2) of the protein interaction network. The protein interaction network was expanded to four levels such that the proteins on each level were curated as interacting with proteins on the previous level (Fig. 1b). Only proteins that were expressed in lung tissue (GTEx^28^) were included in the expanded protein interaction network (severe; n = 462 proteins; and hospitalised; n = 720 proteins; Supplementary Table S3a and b). For replication purposes, the process was repeated using the PROPER-seq protein interaction dataset^32^. In comparison to STRING^31^, PROPER-seq is restricted to empirically captured protein–protein interactions^32^.

**Figure 2:**
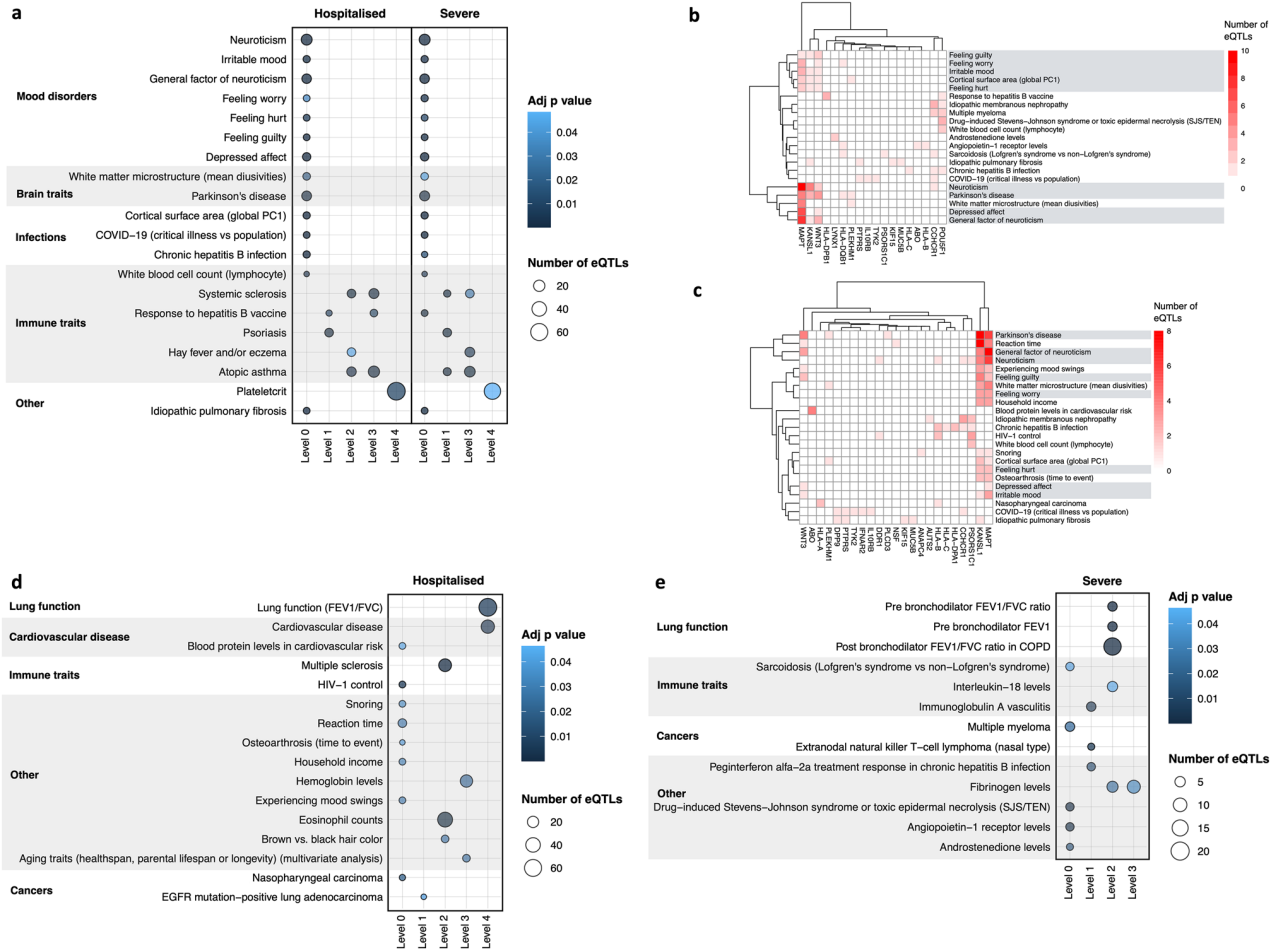
Protein interaction network analysis identifies associations between SARS-CoV-2 and complex traits. The protein interaction network analysis (Fig. 1b) was applied to lung tissue using genes (i.e. severe = 104; hospitalised = 123; Supplementary Fig. 1d; Supplementary Table 2) targeted by SARS-CoV-2 associated eQTLs in this tissue (Supplementary Table 8), and the lung GRN (Supplementary Table 7). Traits that were significant following bootstrapping (*p* ≤ 0.05) were (**a**) shared, (**d**) unique to the hospitalised phenotype, or (**e**) unique to the severe phenotype. Pleiotropic genes are responsible for the trait associations in level 0 for (**b**) the shared severe phenotype and (**c**) the shared hospitalised phenotype. Bi-clustering of the genes x traits was performed using constrained eQTLs for the gene and trait in question. Bubble size is proportional to the total number of eQTLs enriched in each trait, bubble colour is proportional to the adjusted *p*-value (Bonferroni correction) for GWAS trait enrichment.

We parsed all known common SNPs (MAF ≥ 0.05; dbSNP154^33^) through CoDeS3D using lung cell genome structure (Hi-C) and lung tissue gene expression data to identify spatial eQTLs. This analysis generated a lung gene regulatory network (GRN) that consisted of 908,356 spatial eQTLs (731,067 SNPs [MAF ≥ 0.05] and 15,532 genes) that impacted gene expression within lung tissue (“Methods”). We used the lung GRN to obtain eQTLs associated with proteins within levels 1 to 4 of the expanded protein interaction network (Fig. 1b). eQTLs for the genes within each level of the expanded protein interaction network were tested for trait enrichment (hypergeometric test) within the GWAS Catalog. eQTLs were tested for significance within each level independently and were not aggregated across the levels. Bootstrapping (n = 1,000 randomly chosen gene sets of equal size to the severe [n = 104] and hospitalised [n = 123] sets; Supplementary Fig. 1c]) confirmed that 49 of 80 level-specific traits were non-random and unique to SARS-CoV-2 (*p* ≤ 0.05; Supplementary Fig. 2 and Supplementary Table 4). As expected, due to the overlap of SNPs between the hospitalised and severe phenotypes (Supplementary Fig. 1a), a subset of significant trait associations were shared (n = 20; Fig. 2a), unique to hospitalised (n = 16; Fig. 2d), or unique to the severe (n = 13; Fig. 2e) SARS-CoV-2 infection outcomes (Supplementary Table 3a,b).

Inspection of the phenotypes that were associated with the eQTLs for proteins at each interaction network level identifies traits: (1) with obvious relevance (e.g. lung function); (2) that support epidemiological observations (e.g. cardiovascular disease^9^, idiopathic pulmonary fibrosis^34^, mood disorders^35^ and Parkinson’s disease^36^); and (3) that have not yet been, or are weakly implicated in SARS-CoV-2 infection outcome (e.g. and immunoglobulin A vasculitis). Among all 55 significant (*p* ≤ 0.05) traits identified using the STRING-informed protein interaction network, 33 were replicated using a network of protein interactions captured within human embryonic kidney, T lymphocyte, and endothelial cells (PROPER-seq^32^; Supplementary Fig. 3; Supplementary Table 4c,d).

Index level genes that have eQTLs associated with other traits are, by definition, pleiotropic. Seven of the 21 index level traits, for both SARS-CoV-2 phenotypes, were mood disorders (Fig. 2a). The eQTLs associated with the index level mood disorders are associated with *MAPT, KANSL1* and *WNT3* transcript levels (Fig. 2b–c). These genes, in combination with *PLEKHM1* and *HLA-DQB1* are also associated with the GWAS Catalog trait, “Parkinson’s disease” (level 0; Fig. 2a–c). The trait-associated eQTLs (n = 34) that regulate *MAPT* are located across a 1 Mb locus on chromosome 17 (Supplementary Fig. 4). This is consistent with the existence of multiple trait-specific regulatory elements for *MAPT* within chromosome 17q21.31.

“Cardiovascular disease” was significantly associated with the hospitalised (adj *p* = 3.96 × 10^–3^) phenotype within lung tissue, following bootstrapping (Fig. 2d). There was a total of 32 eQTLs and 34 genes enriched for “cardiovascular disease” in the lung interaction network (Supplementary Fig. 5; Supplementary Table 3b). Of the 34 genes, *NOS3, ADK, ACE, AGT* and *PIK3CB* were identified as being druggable targets^37^ (Supplementary Table 5), however the impact of therapeutics on the risk of cardiovascular disease associated with SARS-CoV-2 remains unknown.

Traits affecting lung function share molecular interactions with the SARS-CoV-2 infection phenotypes (Fig. 2d–e). However, the hospitalised phenotype was associated with lung function (FEV1/FVC; Fig. 2d; Supplementary Table 4b)^38^. The eQTLs responsible for this hospitalised phenotype-specific lung function association were linked to 55 genes (Supplementary Fig. 6; Supplementary Table 3b). By contrast, the severe SARS-CoV-2 phenotype was associated with traits that are typically recognised as having greater impact on lung function, e.g., “chronic obstructive pulmonary disorder” (Fig. 2e; Supplementary Table 4a). The severe lung function traits were due to eQTLs targeting *PSMA4* and *CHRNA3* (Supplementary Fig. 6f-g; Supplementary Table 3a). Chronic obstructive pulmonary disorder is an epidemiologically verified comorbidity for severe SARS-CoV-2 infection^8^.

### Tissue specific regulatory roles reveal epidemiologically verified SARS-CoV-2 comorbidities and predispositions

**Figure 3:**
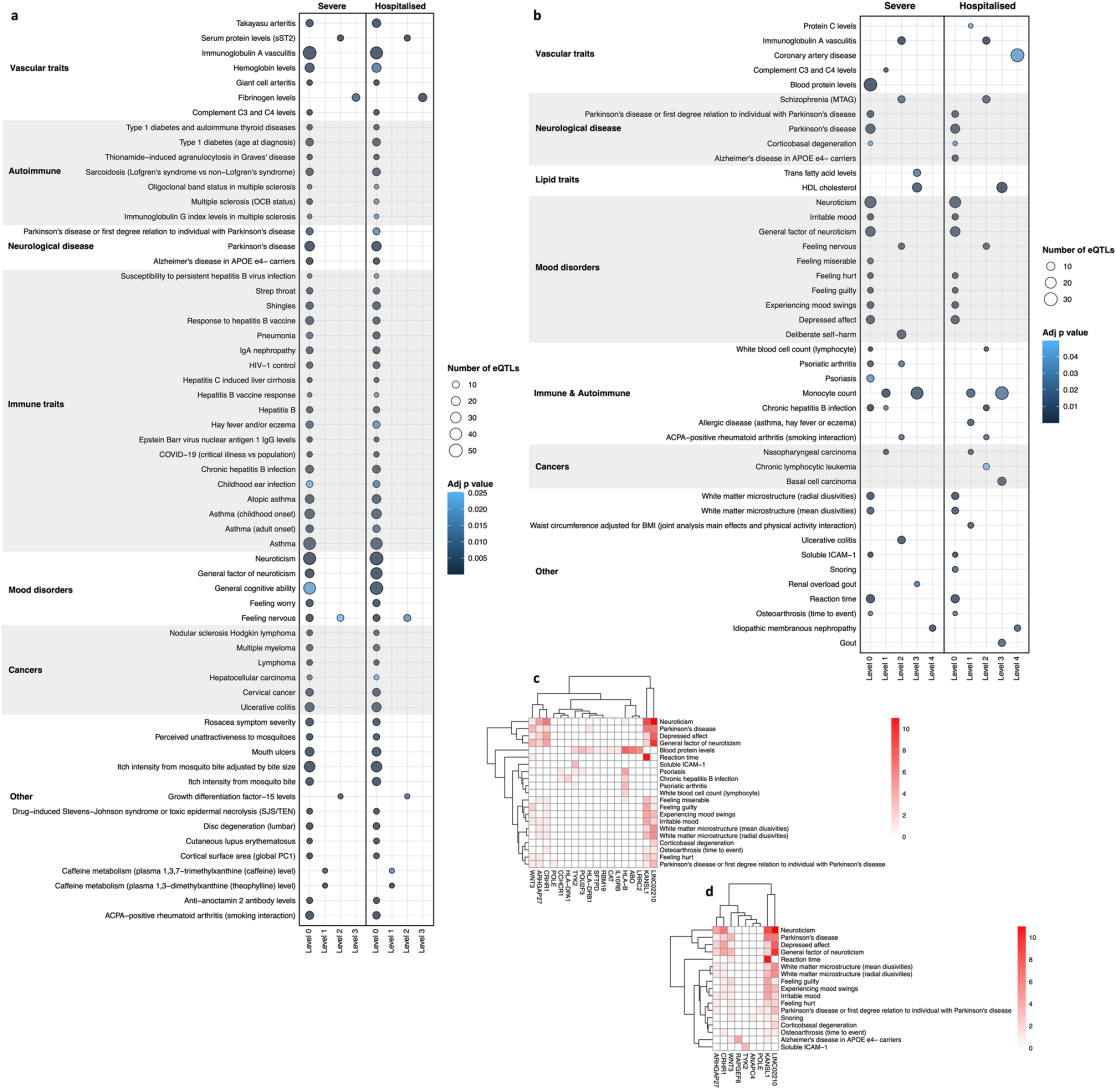
Additional traits are associated with SARS-CoV-2 infection severity in blood and brain. The protein interaction network analysis (Fig. 1b) was applied to whole blood and brain tissues using genes (i.e. severe = 206; hospitalised = 214 in blood, severe = 35; hospitalised = 38 in brain; Supplementary Fig. 1d; Supplementary Table 6) targeted by SARS-CoV-2 associated eQTLs in these tissues (Supplementary Table 6), blood^39^ and brain GRNs (Supplementary Table 7). Blood tissue traits identified from the STRING protein interactions that were (**a**) shared across both severe and hospitalised phenotypes; and (**b**) were shared and observed in the brain. Only traits that were significant following bootstrapping (*p* ≤ 0.05) are shown. Bubble size is proportional to the total number of eQTLs enriched in each trait, bubble colour is proportional to the adjusted *p*-value (Bonferroni correction) for GWAS trait enrichment. The heatmaps highlight genes that are associated with the level 0 traits in the severe (**c**) and hospitalised (**d**) phenotypes in the brain.

SARS-CoV-2 hospitalisation and death^13^ have been epidemiologically linked to obesity and diabetes^10,11^. Neither obesity nor diabetes were identified as being comorbid with infection severity in our analysis of the lung (Fig. 2). However, gene regulation is tissue specific^19,20^ and we hypothesised that the comorbid effects associated with these traits are mediated through other organ(s). Genes targeted by spatially constrained eQTLs were identified (FDR ≤ 0.05) within whole blood and brain cortex using 5,594 SNPs that were associated with SARS-CoV-2 hospitalisation or severe phenotype (Supplementary Fig. 1d; Supplementary Table 6). GRNs for blood^39^ and brain cortex (1,050,155 spatial eQTLs involving 862,964 SNPs and 14,428 genes; Supplementary Table 7) were generated. There were 111 and 43 traits associated (FDR < 0.05) with the SARS-CoV-2 protein interaction network within blood and brain tissue, respectively, following bootstrapping (Fig. 3; Supplementary Fig. 7; Supplementary Table 8). “Type 1 diabetes and autoimmune thyroid diseases” (adj *p* = 3.87 × 10^–4^) and “Type 1 diabetes (age at diagnosis)” (adj *p* = 1.76 × 10^–11^) were significantly associated with the SARS-CoV-2 severe and hospitalisation phenotypes in whole blood tissue (Fig. 3a). These associations were replicated in our analysis of protein interactions derived from PROPER-Seq (Supplementary Figs. 8 and 9; Supplementary Table 8e and g). There are 14 eQTLs and 27 pleiotropic genes, located within the HLA region on chromosome 6, that are associated with “Type 1 diabetes (age at diagnosis)” across both phenotypes in blood (Supplementary Table 3c and d; Supplementary Fig. 10). This is concordant with the major genetic susceptibility determinants for Type 1 diabetes^40^.

We compared the multimorbid traits that were significantly associated, following bootstrapping, with SARS-CoV-2 infection severity across the lung, blood, and brain GRNs (Supplementary Fig. 11a). We identified 471 eQTLs regulating 230 genes enriched for 7 traits (e.g. Parkinson’s disease), which were shared across these tissues (FDR ≤ 0.05; Supplementary Fig. 11b; Supplementary Table 3g). However, whilst the traits are shared, distinct tissue-specific eQTL and gene profiles are responsible for the enrichment of each trait (Supplementary Fig. 11b). Notably, among the unique traits, 14 of the 39 ‘blood traits’ that were associated with the hospitalised phenotype (e.g. cholesterol and fatty acid measures, and serum metabolites in chronic kidney disease) were enriched for eQTLs targeting the *FADS2-FADS1* genes (Supplementary Fig. 12).

### Identification of shared risk for cardiovascular disease factors and SARS-CoV-2 infection

Cardiovascular disease is a known risk factor for acute and post-acute SARS-CoV-2 aetiology^9^. Coronary artery disease (CAD) was associated with both SARS-CoV-2 phenotypes in blood prior to bootstrapping (adj *p* = 3.20 × 10^–3^ and 4.08 × 10^–4^ hospitalised and severe, respectively; Supplementary Table 8a and c). Similarly, CAD was associated with the severe phenotype (adj *p* = 0.004; Supplementary Table 8i) in the coronary artery prior to bootstrapping, but not following (Supplementary Figs. 13 and 14), indicating the association in these tissues may not be unique to SARS-CoV-2, however, still statistically and biologically relevant based on epidemiological studies^9^. CAD remained associated with the hospitalised phenotype in brain following bootstrap (adj *p* = 0.03; Fig. 3b; Supplementary Table 8d).

The CAD-association in brain (Fig. 3b; Supplementary Table 8d) was due to 30 spatially constrained eQTLs and 18 genes, which formed 8 protein clusters and 124 proteins within the expanded protein interaction network (Supplementary Fig. 15; Supplementary Table 9a). The genes (e.g. *ERBB4, NOTCH4, HSD17B12)* and pathways (e.g. ErbB signaling pathway [*p* = 9.36 × 10^–6^]; fatty acid metabolism [*p* = 2.16 × 10^–7^]) have recognised relevance to CAD^41^ and SARS-CoV-2^42^. Notably, one eQTL we identified as regulating *ERBB4* within the brain regulatory map has not been mapped to *ERBB4* by GWAS (Supplementary Table 9b).

Traits known to increase the risk of cardiovascular events (i.e. Takayasu arteritis^43^ [hospitalised adj *p* = 9.51 × 10^–9^; severe adj *p* = 0.001], giant cell arteritis^44^ [hospitalised adj *p* = 7.66 × 10^–5^; severe adj *p* = 4.89 × 10^–5^], immunoglobulin A vasculitis^45^ [hospitalised adj *p* = ; severe adj *p* = 1.89 × 10^–76^]) and clotting factors (i.e. fibrinogen levels^46^ [hospitalised adj *p* = 6.30 × 10^–5^; severe adj *p* = 0.009]) were associated with both phenotypes in blood (Fig. 3a; Supplementary Table 8a and c), brain (i.e. immunoglobulin A vasculitis [hospitalised adj *p* = 4.41 × 10^–6^; severe adj *p* = 2.23 × 10^–8^]; Fig. 3b) and severe only in the lung (i.e. immunoglobulin A vasculitis [adj *p* = 0.007], fibrinogen levels [adj *p* = 0.03]; Fig. 2e).

## Discussion

This study integrated a protein interaction network with tissue-specific gene regulatory networks to identify comorbidities and predispositions of SARS-CoV-2 infection outcomes, and the mechanisms that potentially link them, without a priori assumptions. The analysis identified known comorbid traits such as CAD, type 1 diabetes, mood disorders and asthma etc. Evidence for genetic predispositions for traits that have not previously been associated or have only been weakly associated with SARS-CoV-2 was also obtained (i.e., Parkinson’s disease, Alzheimer’s disease, Hirschsprung disease and inflammatory bowel disease). Collectively our results support the potential for a much greater post-acute SARS-CoV-2 burden if these genetic predispositions are realised.

The pathway and network-based approach we used anchors the convergence of diseases in their shared genetic aetiology. There are two key implications of this new understanding of the genetic and biophysical interactions between the complex conditions and SARS-CoV-2 infection. Firstly, therapeutic stratification of acute and post-acute SARS-CoV-2 patients according to genetically defined comorbidities is possible by analysing the individualised combined genetic burden for SARS-CoV-2 infection outcome and comorbidities. Secondly, therapeutics that address the comorbidities, and thus potentially reduce the impacts of the interactions with SARS-CoV-2 infection, may be clinically viable when applied in individuals who have the predisposing genetic burden.

The discovery-based protein interaction network approach we developed has uncovered putative mechanisms for comorbid and genetic predispositions for traits associated with SARS-CoV-2. However, this study has several limitations. (1) Study cohorts within the GWAS catalogue are biased to participants of European descent. (2) The identification of traits is limited to those that were listed in the GWAS Catalog (02-12-2021). For example, the COVID-19 HGI variants were not listed in the GWAS Catalog when this analysis was performed. (3) We were limited to the analysis of common genetic variants (MAF ≥ 0.05). The inclusion of rare variants, with larger effect sizes, may possibly impact on additional pathways with greater phenotypic consequences. (4) We did not include epigenetic data, which captures environmental interactions, within our analyses. For instance, we have not considered the downstream effects of changes to transcription factor target information or transcript levels on gene expression (5) The protein interaction networks were dependent upon curated protein interaction data from STRING and PROPER-seq. It is likely that these datasets do not capture all biologically relevant protein interactions. Finally, we did not obtain protein interaction, spatial genome [Hi-C], and gene expression data from an identical sample. Therefore, inter-sample variation between the different datasets will impact the analysis.

The population controls used in the COVID-19 HGI consortium were individuals without knowledge of SARS-CoV-2 infection or COVID-19 status^4^. Although this definition of population controls may lead to biased effect size estimates if some of these individuals were exposed to the virus and became infected with SARS-CoV-2 or developed severe COVID-19, we and the COVID-19 Host Genetics Initiative consortium acknowledge this limitation. However, the COVID-19 Host Genetics Initiative conducted sensitivity analyses and determined that the use of population controls in infectious disease host genetic studies is a valid approach^4^.

Several of the target genes we identified within the index level are novel due to both a) the incorporation of variants with suggestive significance and b) spatial regulatory information. For example, *SMARCA4* was identified as being targeted by lung specific eQTLs (rs10416073, rs7247198) in the severe phenotype (within the limitations COVID-19 Host Genetics Initiative definition). Notably, this gene was not identified as a target in the SARS-CoV-2 GWAS^2,4,47^. However, *SMARCA4* was identified by CRISPR screen to be the second strongest SARS-CoV-2 pro-viral gene after *ACE2*^48^. We contend that the convergence of results from candidate gene and population studies supports the putative biological importance of our expanded findings, compared to the SARS-CoV-2 GWAS studies^1–7^.

We identified tissue-specific pleiotropy between SARS-CoV-2 infection and the genetic risk for Parkinson’s disease, neurological conditions, and mood disorders. Parkinson’s disease was identified as being causally related to SARS-CoV-2^4^. Whilst the biological relevance of this relationship is unclear, we identified a total of 26 variants and 28 genes (e.g. *MAPT*, *CRHR1,* and *KANSL1*)^49^ across all tissues tested that are associated with this link. This association was driven predominantly by HLA region (i.e. 6p21) variants and the 17q21.31 locus. Consistent with our findings, the 17q21.31 locus has been identified as linking SARS-CoV-2 and Parkinson’s disease^15^, likely driven by the recognised inversion in this region. We have expanded on the proposed 17q21.31 linkage between SARS-CoV-2 and Parkinson’s disease by identifying 4 variants and 2 pleiotropic genes (i.e. *TLK1 and FDFT1*) in blood, located outside 17q21.31 and 6p21, that are also associated with both traits. Moreover, the integration of spatial constraints in the identification of tissue-specific regulatory connections (i.e. constrained eQTLs), reduced the overall number of traits and genes that were associated with the pleiotropic 17q21.31 locus^15^. Whilst the long-term significance of SARS-CoV-2 infection and Parkinson’s disease onset and severity remains inadequately understood, this is an area of concern^36^. Notably, the 1918 Spanish flu (influenza A H1N1 virus) pandemic resulted in an increase in the incidence of Parkinson’s disease^50^. Therefore, we contend that the genetic architecture and protein interactions we identified may represent high-value therapeutic targets to affect the causal relationship^4^ and reduce long-term increases in the incidence of Parkinson’s disease following SARS-CoV-2 infection.

Consistent with epidemiological observations^10–12^, we identified type 1 diabetes (age at diagnosis) as being associated with the severe and hospitalised phenotypes, as defined by the COVID-19 Host Genetics Initiative. This association was due to 27 pleiotropic genes (e.g. *NOTCH4*). Collectively, these results suggest several putative mechanisms that may link type 1 diabetes and SARS-CoV-2 infection^51^.

Cardiovascular disease burden increases according to severity of SARS-CoV-2 infection^9^. However, the mechanism by which this increase occurs is unknown. In the hospitalised phenotype, we identified 34 genes and 32 eQTLs enriched for cardiovascular disease in the lung protein interaction network and 18 genes and 30 eQTLs enriched for the CAD-association in the brain protein interaction network. We have reproduced and expanded on the known genetic correlation between CAD and SARS-CoV-2^4^ by including tissue specific^19,20^ and spatial^23,24^ regulatory mechanisms in our analysis. The proteins encoded by CAD-associated genes in brain (e.g. *ERBB4* [eQTL rs582384]) functioned within pathways (e.g.“ErbB signaling pathway”) that are activated in CAD, exerting disease mitigation and regenerative effects, and preventing pathological processes (i.e. atherosclerosis) that trigger CAD^41^. Therefore, since the variants we identified are found in the germline, we contend that a genetic predisposition for CAD can amplify the risk of adverse SARS-CoV-2 outcomes. Moreover, in individuals who develop CAD following SARS-CoV-2 infection, the infection activates an existing, albeit unrecognised, genetic predisposition for CAD. ERBB4 is found here to be interacting significantly with NGR1 (NGR-1), an agonist of the ErbB4 receptor. The NRG-1/ErbB4 signalling system is critical for the mitigation of heart failure, an outcome of late-stage CAD. Circulating NRG-1 levels are inversely related with the severity of CAD lesions, it reduces the magnitude of ischemic heart and brain injury, and inhibits atherogenesis via suppression of macrophage cell formation^41^. NRG-1 also inhibits cellular senescence, a key contributor to atherosclerosis, via ErbB4^52^. Clinical trials of recombinant NRG-1 acting via ErbB4 successfully improved overall survival in a cohort of 1,600 patients with heart failure^52^.

In conclusion, the network approach we developed here anchors known SARS-CoV-2 comorbidities and previously undescribed genetic predispositions in a shared genetic aetiology. In so doing, it identifies molecular insights, and potential therapeutic targets. Collectively, these findings pave the way for patient stratification, not simply based on their visible comorbidities, but through an in depth understanding of genetic impacts on traits that collectively alter an individual’s predisposition to acute and post-acute SARS-CoV-2 infection outcomes.

## Methods

### Genetic variants used in this study

Genome-wide association study (GWAS) data for SARS-CoV-2 clinical phenotypes was obtained from the Covid-19 Host Genetics initiative (COVID-19 HGI)^47^. Single nucleotide polymorphisms (SNPs) for the hospitalised versus population and severe (hospitalised AND death or respiratory support) versus population (*p*-value threshold of 1 × 10^–5^) cohorts were obtained from COVID-19 HGI release 6 (https://www.covid19hg.org/results/r6/; Supplementary Table 1). Full summary statistics and details from COVID-19 HGI are available at https://app.covid19hg.org/^47^.

### Assigning putative transcriptional functions to SARS-CoV-2 SNPs

Severe and hospitalised SARS-CoV-2 associated SNPs were analysed separately using CoDes3D^29^ to identify phenotype-specific spatially constrained expression quantitative trait loci (eQTLs) and their target genes (Supplementary Table 2a and b). Phenotype-specific (i.e. hospitalised or severe) spatial connections for each SNP-gene pair were identified from Hi-C chromatin contact data derived from human lung primary tissue^30^, blood (peripheral blood B cells, peripheral blood CD4^+^ T cells, peripheral blood CD8^+^ T cells^53^, peripheral blood T cells^54^), brain (dorsolateral prefrontal cortex cells^30^) and the coronary artery (smooth muscle cells^55^). To identify which SNPs are eQTLs, the SNP-gene pairs were used to query lung, whole blood, brain cortex and the coronary artery within the GTEx database^28^. Multiple testing was corrected using the Benjamini–Hochberg procedure (FDR < 0.05) and interactions were kept if the logarithm of allelic fold change (log_aFC) ≥ 0.05^29^. eQTL and gene chromosome positions were annotated according to human reference genome GRCh38/hg19.

### LD analysis

LD analysis was conducted for eQTL-gene combinations using LDLink 4.0 LDMatrix Tool (https://ldlink.nci.nih.gov/?tab=ldmatrix). Parameters included: SNP rsID numbers from dbSNP154^33^; genotyping data from phase 3 (version 5) of the 1000 Genome Project; European population.

### Generation of gene regulatory networks

We generated gene regulatory networks (GRNs), which included all spatially constrained eQTLs for all known SNPs (MAF ≥ 0.05; dbSNP154^33^) for lung, whole blood (dbGaP accession: phs000424.v8.p2; approved project number: #22937) and brain cortex (GTEx v 8.0)^28^. SNPs were screened through CoDes3D one chromosome at a time. Multiple testing was corrected using the Benjamini–Hochberg procedure (FDR ≤ 0.05) and interactions were kept if the logarithm of allelic fold change (log_aFC) ≥ 0.05^29^.

### Protein–protein interaction network analysis

Curated protein–protein interaction data were obtained from STRING (https://string-db.org). STRING was mined using lists of genes targeted by spatially constrained eQTLs and the following parameters: experiments, text mining, co-expression and databases, species limited to “Homo sapiens”, and an interaction score ≥ 0.7.

Experimentally validated protein interaction data was also obtained from the protein–protein interaction sequencing (PROPER-Seq) tool database (v1.0; https://genemo.ucsd.edu/proper/). Protein interactions were obtained from HEK293T cells, Jurkat cells, and human umbilical vein endothelial cells (HUVECs). Genes targeted by spatially constrained eQTLs were imputed to the PROPER-Seq tool to discover additional cell-line specific protein–protein interactions.

### Expanded protein–protein interaction network analysis

The expanded protein–protein interaction network analysis first takes genes of interest (i.e. the SARS-CoV-2 genes identified by CoDes3D), then parses these genes to STRING^31^, or PROPER-Seq^32^ databases, to identify protein interactions (Fig. 1b). The input gene list is assigned as level 0. The proteins in Levels 1 to 4 include proteins for which there are curated interactions with the previous level. Proteins within levels 1 to 4 may, or may not, interact with each other. The genes that encode the proteins that are present within each level of the protein interaction network (0–4) were then mined against the lung, whole blood and brain-specific GRNs to identify all significant (adj *p* ≤ 0.05) spatially constrained regulatory eQTLs that are associated with the genes of interest (Fig. 1c). The spatially constrained eQTLs are tested for enrichment within SNPs associated with GWAS traits within the GWAS catalogue (*p* = 10^–8^). Curated GWAS associations were downloaded from the NHGRI-EBI GWAS Catalogue^38^ on 02-12-2021. Statistically significant eQTL enrichments were determined by hypergeometric distribution analysis (*p* ≤ 0.05), calculated on the total number of spatially constrained eQTLs at each protein interaction network level. Bonferroni correction for multiple hypothesis testing was calculated on the enriched eQTLs using the *p*-value list and the number of tests that were performed^58^. eQTLs with an adjusted *p*-value ≤ 0.05 were selected as being significant.

Bootstrapping analysis (n = 1,000 iterations) was conducted to determine traits identified by the protein interaction network (at all levels) that are uniquely associated with SARS-CoV-2. Genes lists of the same size as the protein interaction network input datasets (i.e. severe = 104; hospitalised = 123 in lung, severe = 206; hospitalised = 214 in blood, severe = 35; hospitalised = 38 in brain, severe = 86; hospitalised = 89 in coronary artery; Supplementary Fig. 1d; Supplementary Table 2 and 6) were generated randomly from GenBank. The protein interaction network analysis pipeline was run on lung, blood, brain, and coronary artery tissues using the random gene lists. The number of shared traits were compiled in a python dictionary and calculated for significance according to frequency (*p* = trait/1000). Traits with *p*-value ≤ 0.05 were deemed to be unique to SARS-CoV-2.

### Functional and pathway enrichment analyses

Gene Ontology (GO) and Kyoto Encyclopedia of Genes and Genomes (KEGG^59–61^) pathway enrichment analysis was conducted using g:Profiler (https://biit.cs.ut.ee/gprofiler/gost) and the Reactome (REAC), WikiPathways (WP), Transfac (TF), mirTarBase (MIRNA), Human Protein Atlas (HPA), CORUM and Human Phenotype Ontology (HP) databases. Pathways and significant terms were selected with the threshold of adjusted *p*-value < 0.05.

### Data visualisation used in this study

R studio (version 1.3.959), and ggplot2^62^, VennDiagram^63^ and UpsetR^64^ R packages were used to visualise results. Cytoscape (version 3.8.2) was used for visualising the STRING network. K-means clustering was performed using the R package pheatmap.

## Supplementary Information

Supplementary Figures.

Supplementary Table 1.

Supplementary Table 2.

Supplementary Table 3.

Supplementary Table 4.

Supplementary Table 5.

Supplementary Table 6.

Supplementary Table 7.

Supplementary Table 8.

Supplementary Table 9.

Supplementary Table 10.

Supplementary Table 11.

## Data Availability

The code and data sources used in the analysis are listed in Supplementary Table 11. All findings, scripts and the reproducibility report are available on github at https://github.com/rkjaros/covid_multimorbidity. All figures and gene regulatory networks are available on figshare (https://doi.org/10.6084/m9.figshare.c.6078462.v1).
