# Supplementary material for: Comorbidity genetic risk and pathways impact SARS-CoV-2 infection outcomes
Source: Sci Rep. 2023 Jun 19;13:9879. doi: 10.1038/s41598-023-36900-z (PMC10279740; doi:10.1038/s41598-023-36900-z)
Supplement: Supplementary file 1 — Supplementary Figures. [file 41598_2023_36900_MOESM1_ESM.pdf]

Additional information

Comorbidity genetic risk and pathways impact SARS-CoV-2 infection outcomes

Rachel K. Jaros[1], Tayaza Fadason[1,2], David Cameron-Smith[3], Evgeniia Golovina[1], Justin M. O'Sullivan[1,2,4,5,6*]

1. The Liggins Institute, The University of Auckland, Auckland 1023, New Zealand.

2. Maurice Wilkins Centre for Molecular Biodiscovery, The University of Auckland, Auckland 1010, New Zealand.

3. College of Health, Medicine and Wellbeing, The University of Newcastle, Callaghan 2308, Australia.

4 MRC Lifecourse Epidemiology Unit, University of Southampton, United Kingdom

5. Singapore Institute for Clinical Sciences, Agency for Science, Technology and Research (A*STAR), Singapore, Singapore

6. Australian Parkinson's Mission, Garvan Institute of Medical Research, Sydney, New South Wales, Australia

*Corresponding senior author.

Extended data - figures

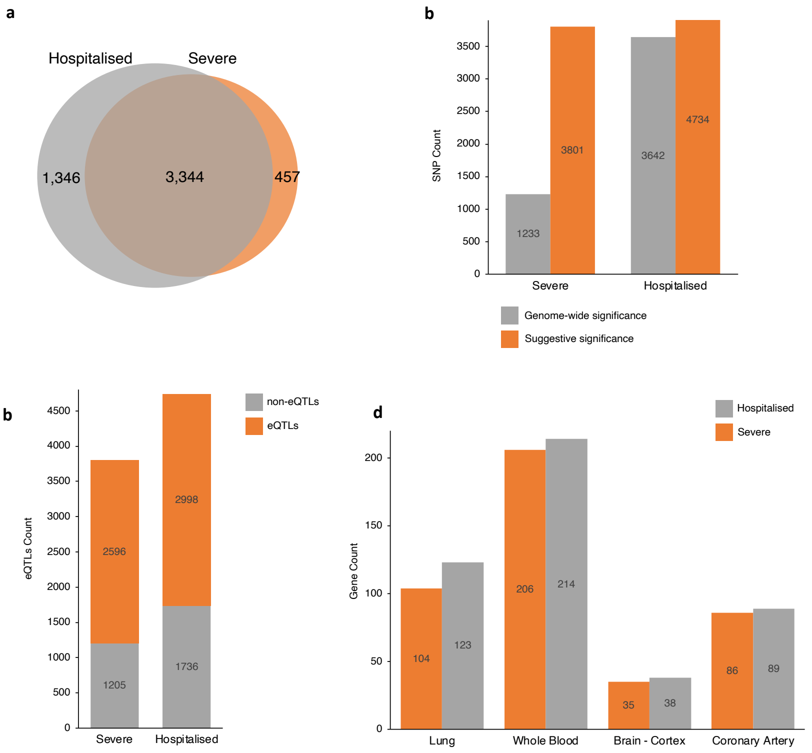

Supplementary Figure 1 l Features of SARS-CoV-2 associated variants and the identified regulatory interactions **(a)** GWAS SNPs shared between hospitalised and severe phenotypes **(b)** Count of SNPs from COVID-19 HGI datasets (hospitalised and severe) that reached genome-wide significance ($p < 5 \times 10^{-8}$) compared to suggestive significance ($p < 1 \times 10^{-5}$). **(c)** Count of eQTLs and non-eQTLs in severe and hospitalised phenotypes in the lung. **(d)** Count of SARS-CoV-2 eQTL target genes (i.e. index level genes in the comorbidity analysis) in lung, whole blood, brain and the coronary artery.

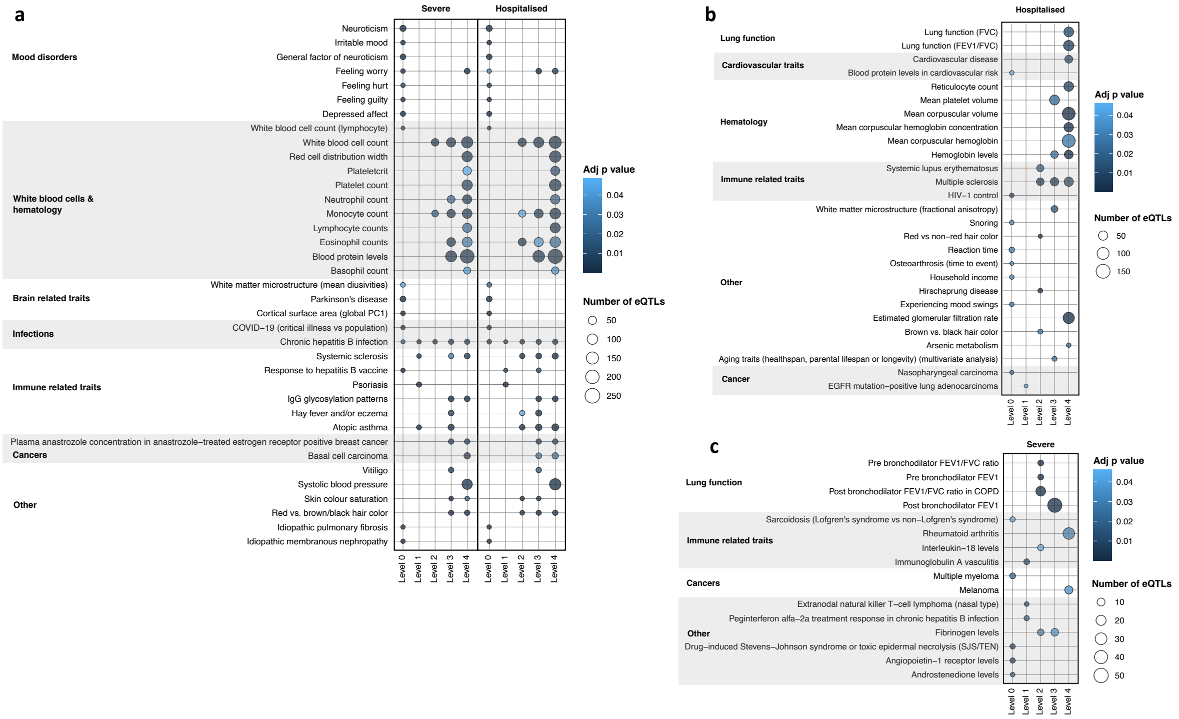

Supplementary Figure 2 | All traits that interact with SARS-CoV-2 eQTL targeted genes within the lung. Graphs include all traits that were identified prior to bootstrapping to complement Figure 2. **(a)** highlights shared traits, **(b)** traits unique to hospitalised phenotype, and **(c)** traits unique to the severe phenotype. Bubble sizes are proportional to the total number of eQTLs for each trait, colour is proportional to the adjusted *p*-value (Bonferroni correction) for GWAS enrichment.

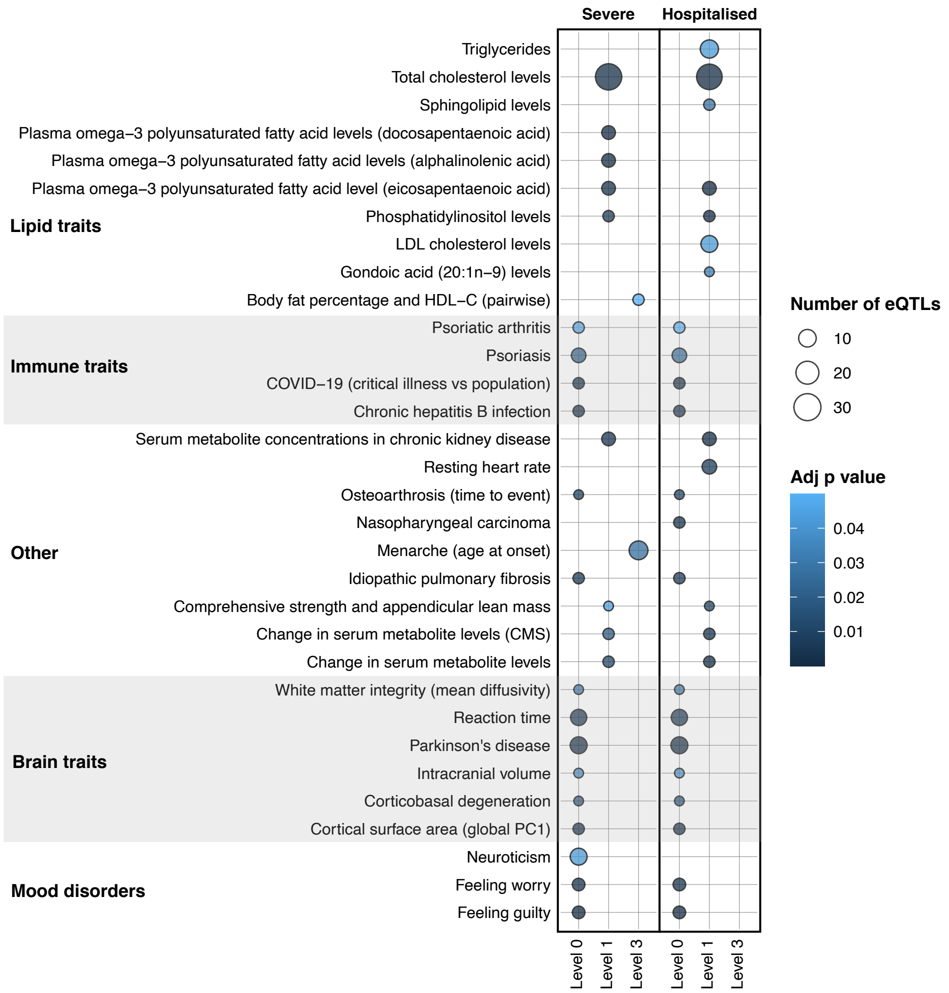

Supplementary Figure 3 | GWAS traits in the SARS-CoV-2 hospitalised and severe phenotypes, which were identified using protein interactions from PROPER-Seq and a lung GRN. Here, the genes targeted by eQTLs within the lung tissue (identified using CoDeS3D) were used as the index level for a protein interaction network defined using protein interaction data from PROPER-Seq[32] (see Methods). The eQTLs for the genes within the extended PROPER-Seq network were identified from the lung GRN. There are 21 significant (adj $p < 0.05$) shared traits following bootstrap. Cholesterol traits (i.e. triglycerides (adj p = 0.04) and LDL cholesterol levels (adj p = 0.04) were associated with the hospitalised phenotype only. By contrast, obesity measures (i.e. body fat percentage and HDL-C (pairwise) (adj p = 0.04)) is associated with the severe phenotype only. Bubble sizes are proportional to the total number of eQTLs for each trait, colour is proportional to the adjusted p-value (Bonferroni correction) for GWAS enrichment.

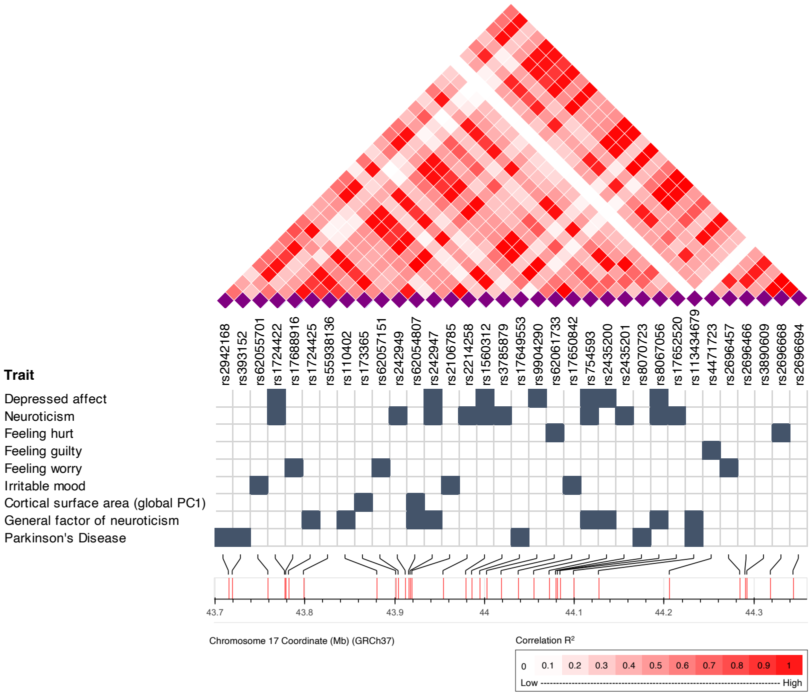

Supplementary Figure 4 | Lung-eQTLs regulating *MAPT*, and their trait associations are spread throughout 17q21.31, consistent with the existence of multiple regulatory elements for *MAPT*. 34 lung-eQTLs associated with mood disorders and Parkinson's disease (Figure 2b and c) regulate *MAPT* (Supplementary Table 6). However, whilst the gene is shared across the severe and hospitalised phenotype and across associated traits, the lung-eQTLs are distinct. Red shading indicates LD correlation R^2 score.

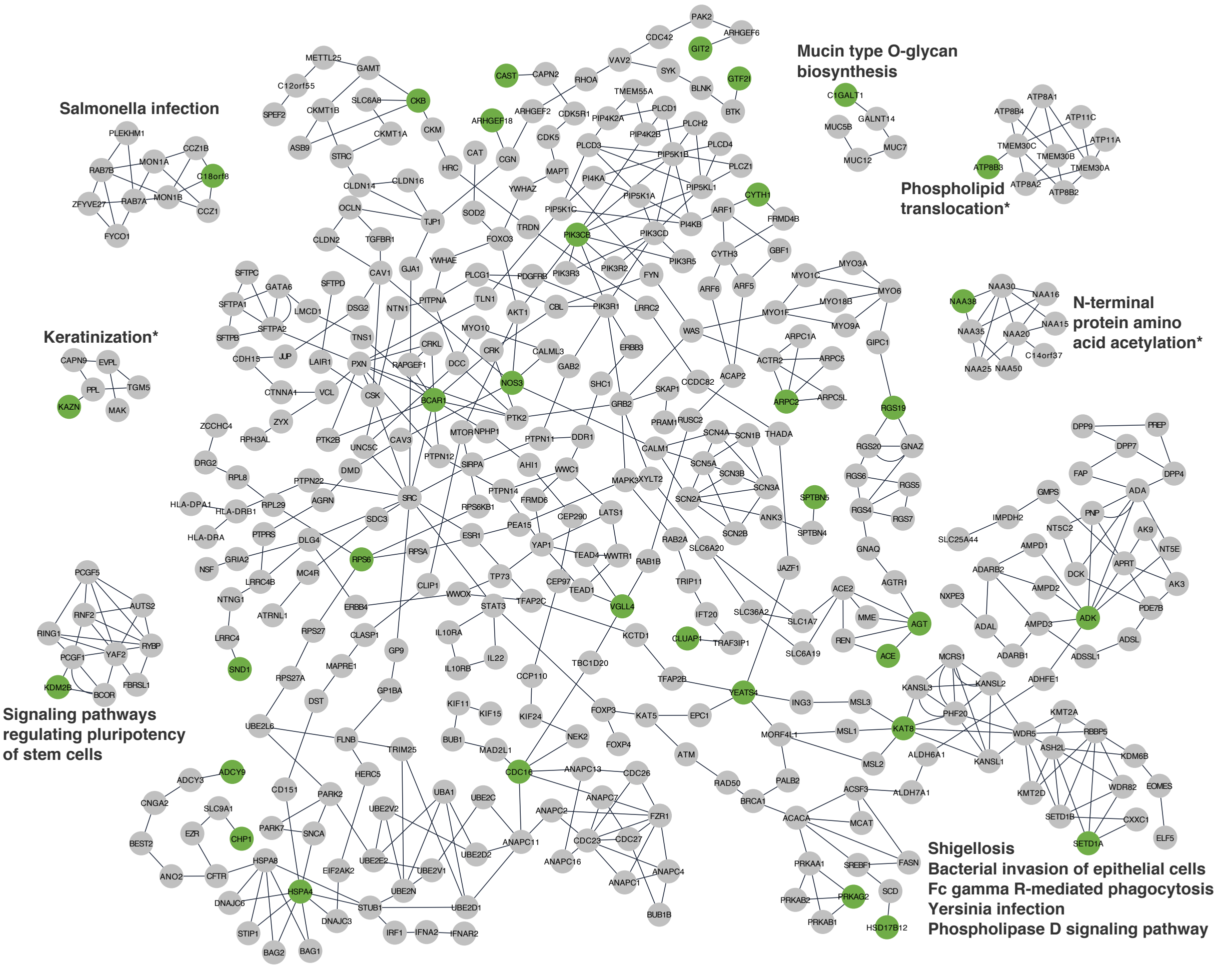

Supplementary Figure 5 | Protein interaction network for lung tissue cardiovascular disease association in the hospitalised phenotype. The protein interaction network shows the hospitalised cardiovascular disease-associated gene products (i.e. proteins at Level 4; Figure 2d) and their interacting partners from Levels 0 to 3. The trait association is driven by lung tissue-specific eQTLs derived from the lung gene regulatory network. The protein network visualises the cardiovascular disease-association as an entire protein network. The level 4 proteins (the encoding genes and their eQTLs) enriched for cardiovascular disease (n = 34) are coloured green. Grey coloured proteins (n = 381) are interacting partners forming the network from level 0 to level 3. There are a total of 34 cardiovascular disease-genes and 32 cardiovascular disease-eQTLs (Supplementary Table 10a) driving this association. The top KEGG pathway(s) or *gene ontology: biological process is listed next to each protein cluster. The genes (e.g. *CKB, ACE, AGT*) and their interacting partners (e.g. *CKM, MME, FEN, ACE2*) have relevance to cardiovascular disease and SARS-CoV-2. Cytoscape (version 3.8.2) was used to visualise the network.

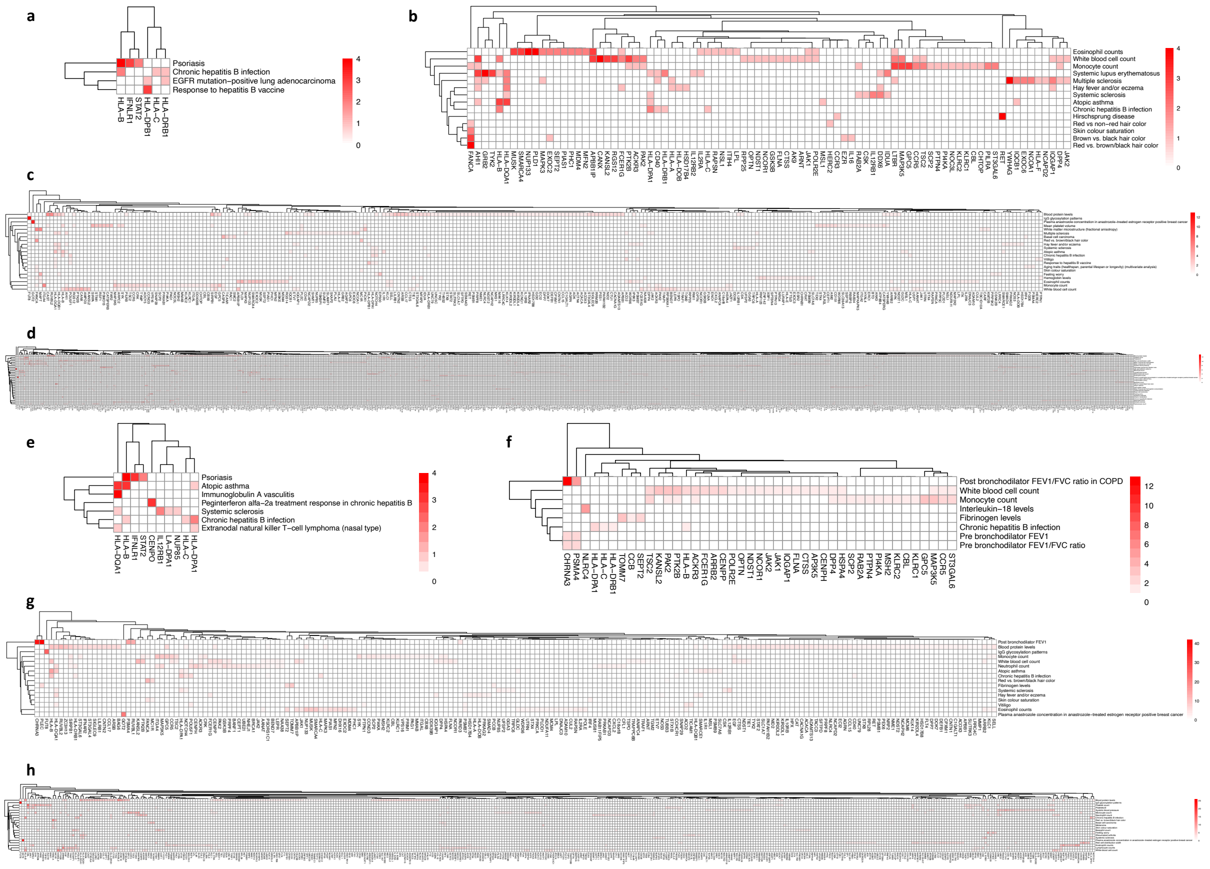

Supplementary Figure 6 | Genes within the network interaction analyses for the hospitalised and severe phenotypes in the lung. Bi-clustering identifies genes and gene clusters that are associated with the significant traits within levels 1 **(a)**, 2 **(b)**, 3 **(c)**, and 4 **(d)** of the hospitalised phenotype and levels 1 **(e)**, 2 **(f)**, 3 **(g)**, and 4 **(h)** of the severe phenotype. The high resolution image is available on figshare (DOI: 10.6084/m9.figshare.20205569)

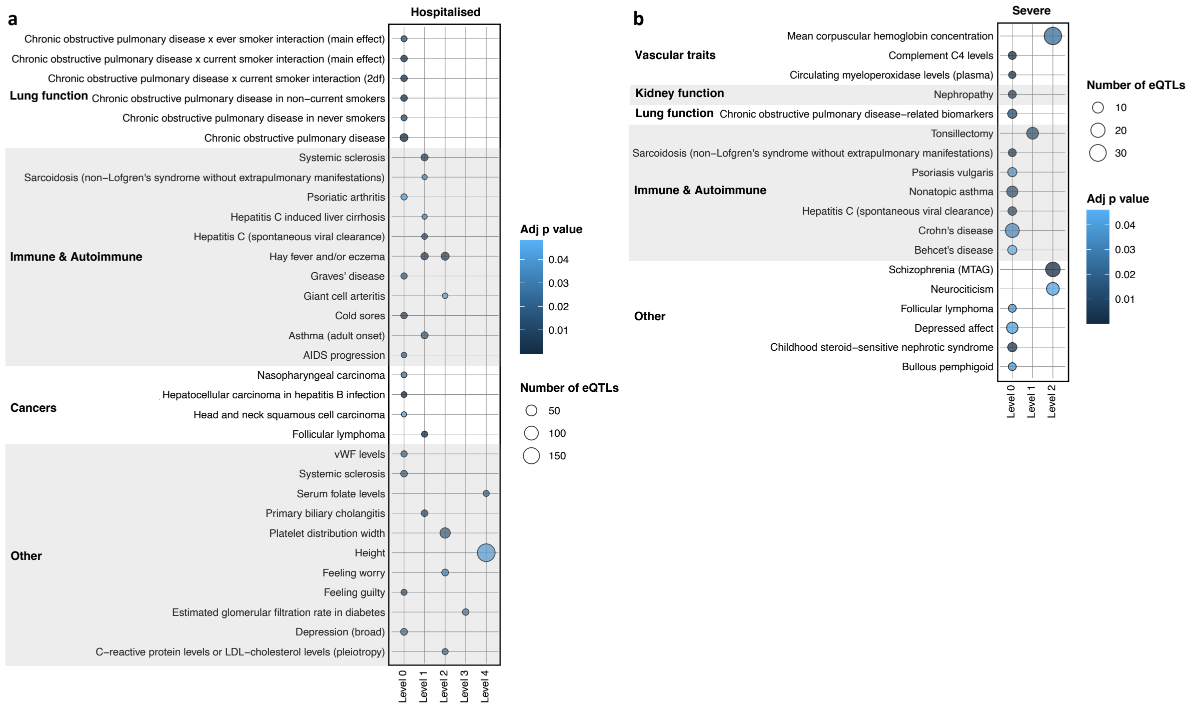

Supplementary Figure 7 | Severe and hospitalised SARS-CoV-2 phenotype trait associations in blood. Graphs include traits unique to **(a)** severe and **(b)** hospitalised phenotypes that were identified from the STRING protein interaction network analysis in whole blood (Figure 3a; Supplementary Table 8). Only traits that were significant following bootstrapping (p ≤ 0.05) are shown. Bubble size is proportional to the total number of eQTLs enriched in each trait, bubble colour is proportional to the adjusted *p*-value (Bonferroni correction) for GWAS trait enrichment.

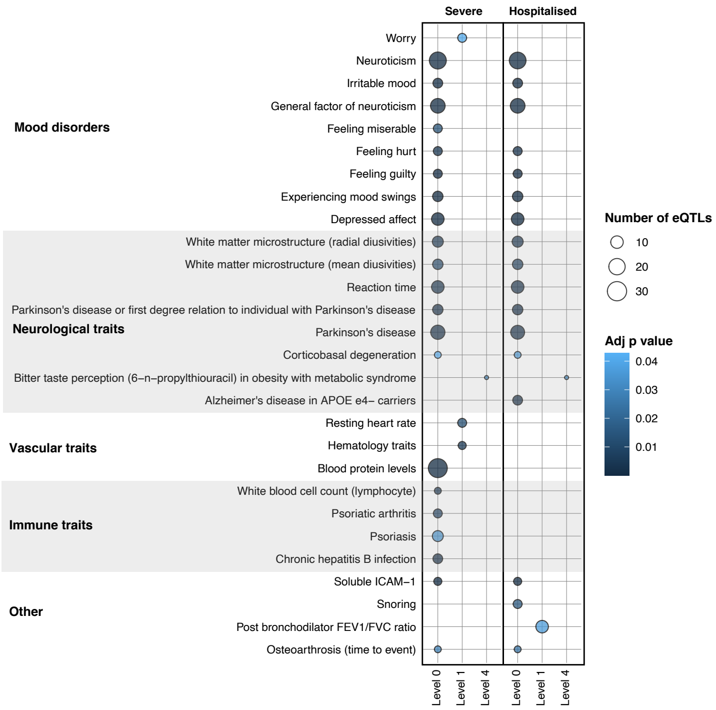

Supplementary Figure 8 | Hospitalised and severe phenotype SARS-CoV-2 trait associations identified using protein interactions from PROPER-Seq in the brain. Graphs include traits that were identified from the PROPER-Seq[32] protein interaction network analysis in brain cortex (Supplementary Table 8). Only traits that were significant following bootstrapping ($p \leq 0.05$) are shown. Bubble size is proportional to the total number of eQTLs enriched in each trait, bubble colour is proportional to the adjusted *p*-value (Bonferroni correction) for GWAS trait enrichment.

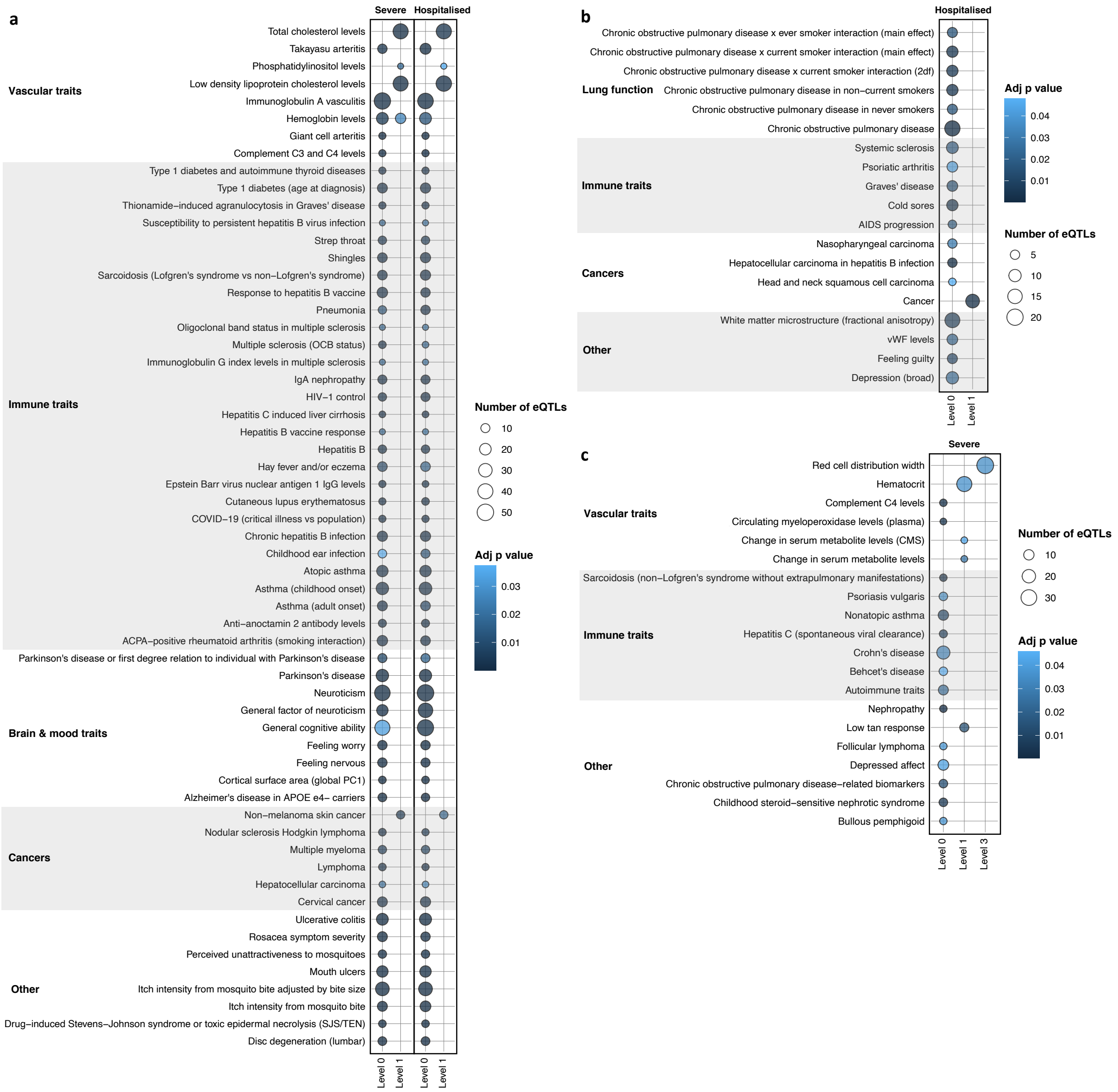

Supplementary Figure 9 | Hospitalised and severe phenotype SARS-CoV-2 trait associations identified using protein interactions from PROPER-Seq in the blood. Graphs include traits that were identified from the PROPER-Seq[32] protein interaction network analysis in whole blood (Supplementary Table 8). Only traits that were significant following bootstrapping (p ≤ 0.05) are shown. Bubble size is proportional to the total number of eQTLs enriched in each trait, bubble colour is proportional to the adjusted *p*-value (Bonferroni correction) for GWAS trait enrichment.

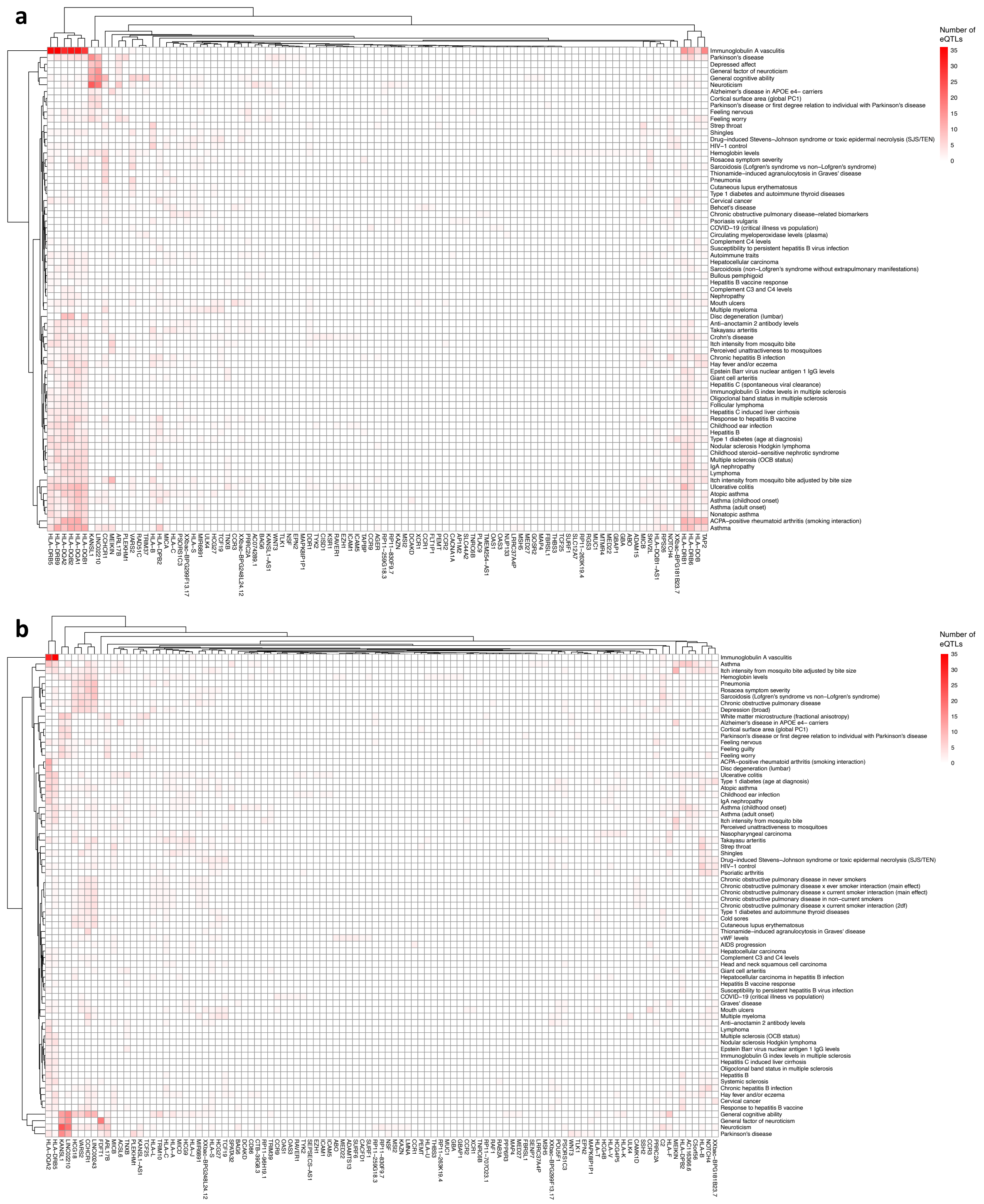

Supplementary Figure 10 | Bi-clustering analysis identifies clusters of traits around specific genes within the network interaction analyses for the hospitalised and severe phenotypes at level 0 in the blood. Heatmaps show genes that are associated with the significant traits at level 0 from the severe **(a)** and hospitalised **(b)** phenotypes in the blood tissue analysis. High resolution images are available on figshare (DOI: 10.6084/m9.figshare.20205575).

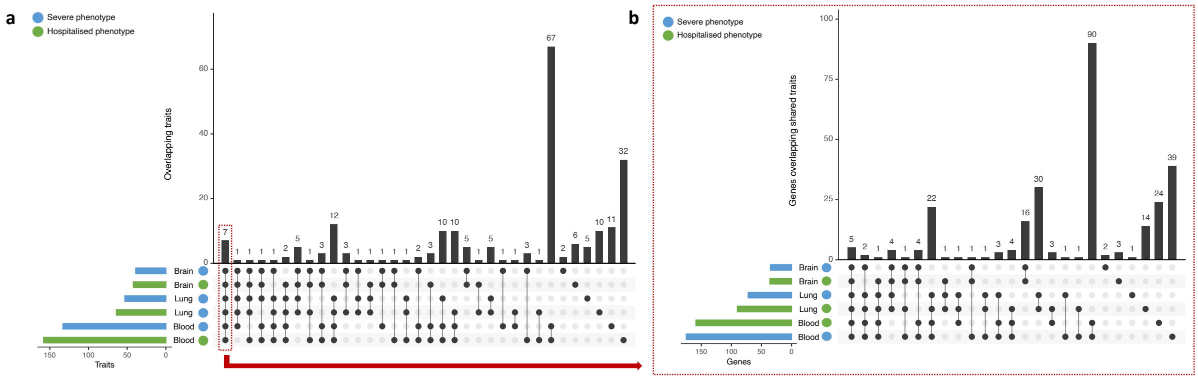

Supplementary Figure 11 | Shared traits are driven by distinct regulatory elements. The upset plot **(a)** compares traits that interact with SARS-CoV-2 hospitalised or severe phenotypes by tissue (i.e. blood, brain, lung). Whilst traits are shared, there is a distinct tissue-specific gene **(b)** profile driving the enrichment for shared traits across all three tissues.

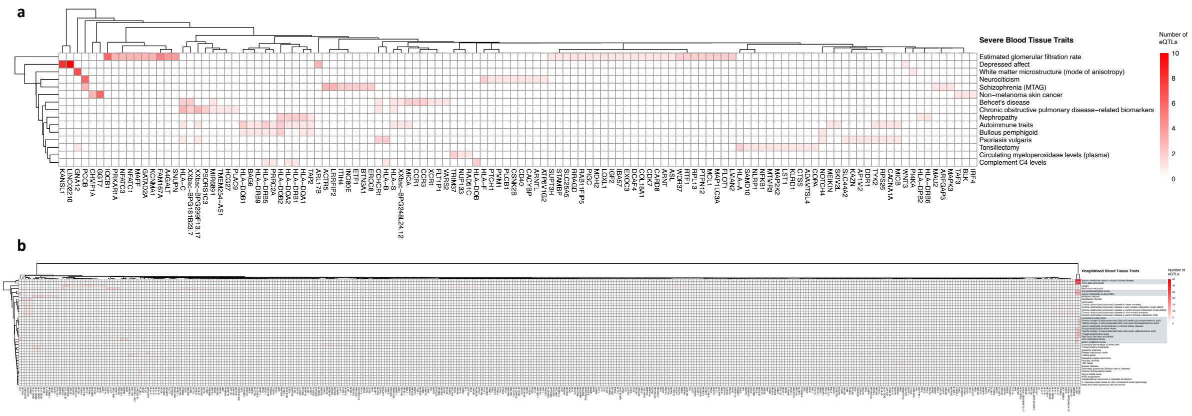

Supplementary Figure 12 | Genes that are associated with the unique traits across all levels in blood in the severe (a) and hospitalised (b) phenotypes. (b) 14 of 39 unique hospitalised blood traits (e.g. Cholesterol and fatty acid measures, and serum metabolites in chronic kidney disease) are being driven by the *FADS2-FADS1* gene cluster. The heatmaps were derived from tissue/phenotype specific data shown in Figure 3a and Supplementary Figure 7. Higher resolution images are available on figshare (DOI: 10.6084/m9.figshare.20205584).

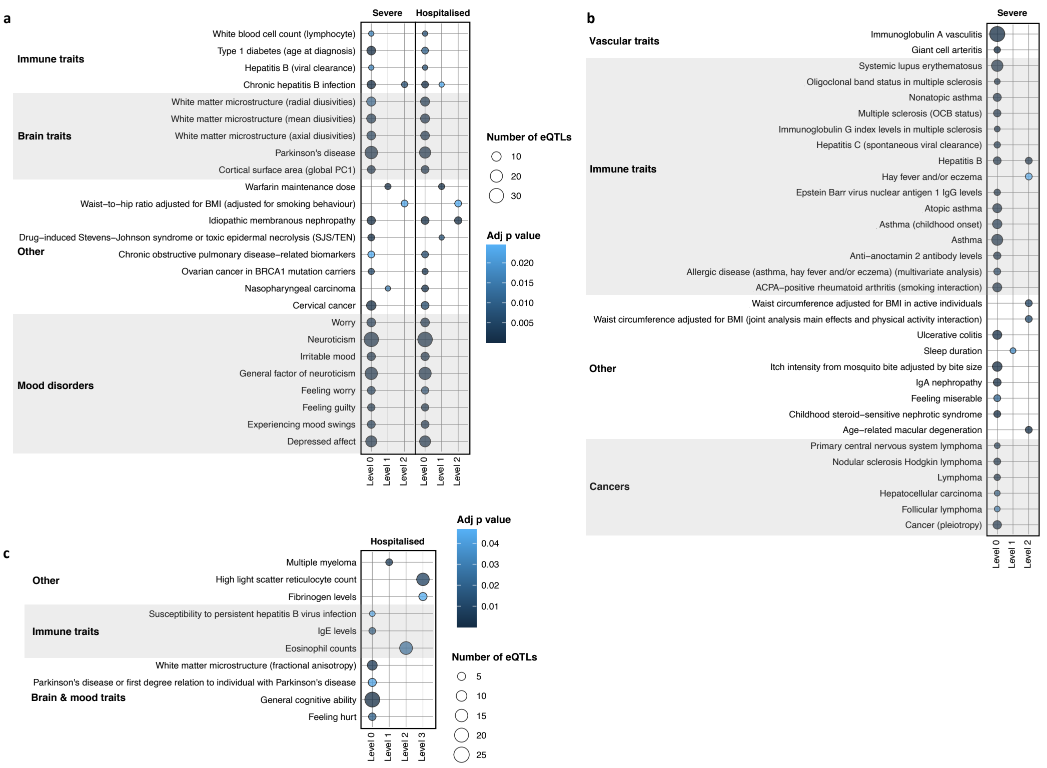

Supplementary Figure 13 | SARS-CoV-2 trait associations in coronary artery tissue using protein interactions from STRING. Graphs include **(a)** shared, **(b)** severe and **(c)** hospitalised phenotype traits that were identified from the STRING derived protein interaction network analysis in coronary artery (Supplementary Table 9i and j). Only traits that were significant following bootstrapping ($p \leq 0.05$) are shown. Bubble size is proportional to the total number of eQTLs enriched in each trait, bubble colour is proportional to the adjusted *p*-value (Bonferroni correction) for GWAS trait enrichment.

a

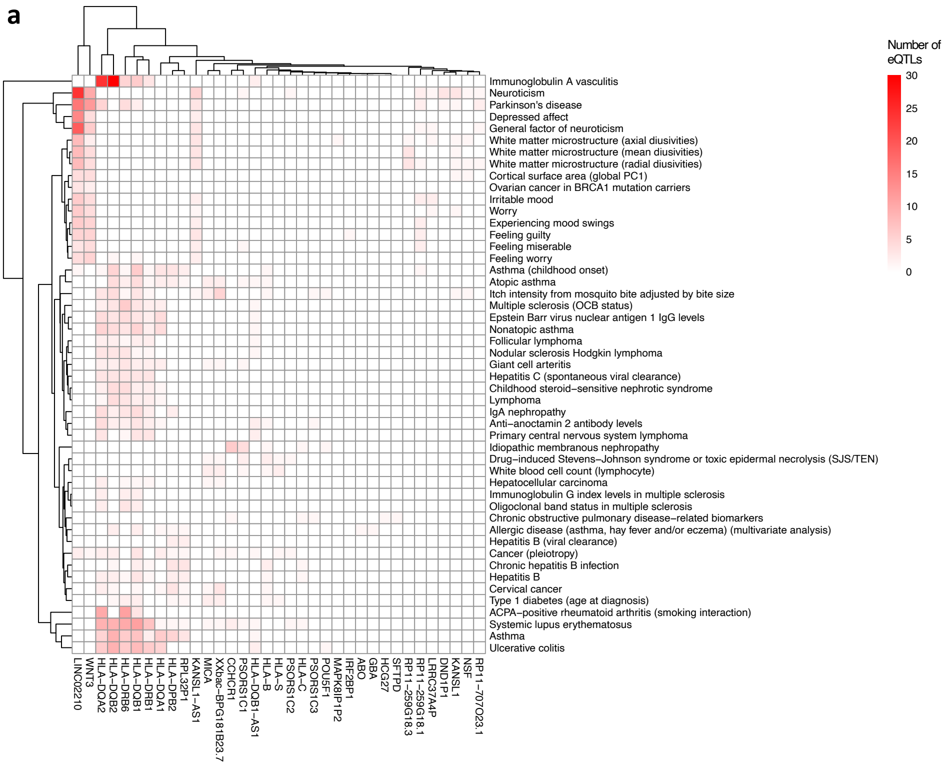

b

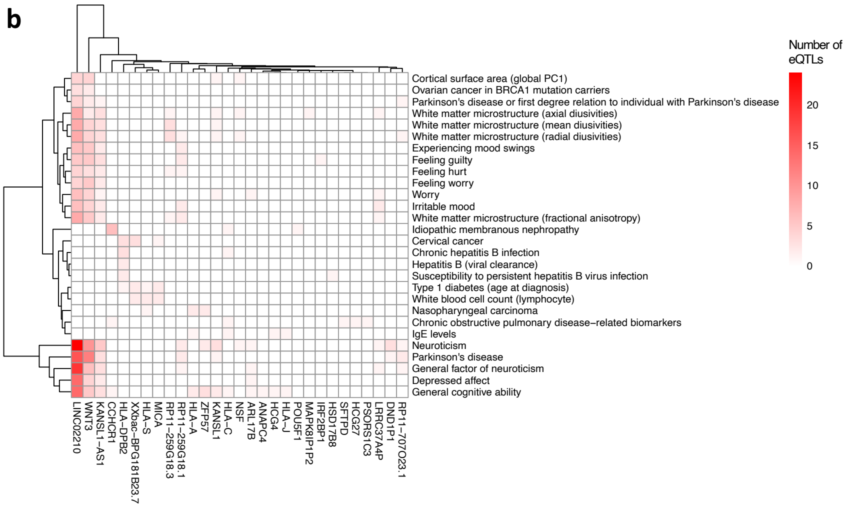

Supplementary Figure 14 | Genes within the network interaction analyses for the hospitalised and severe phenotypes at level 0 in the coronary artery. Heatmaps show genes that are associated with the significant traits at level 0 from the severe **(a)** and hospitalised **(b)** phenotypes in the coronary artery tissue analysis. The high resolution image is available on figshare (DOI: 10.6084/m9.figshare.20205608).

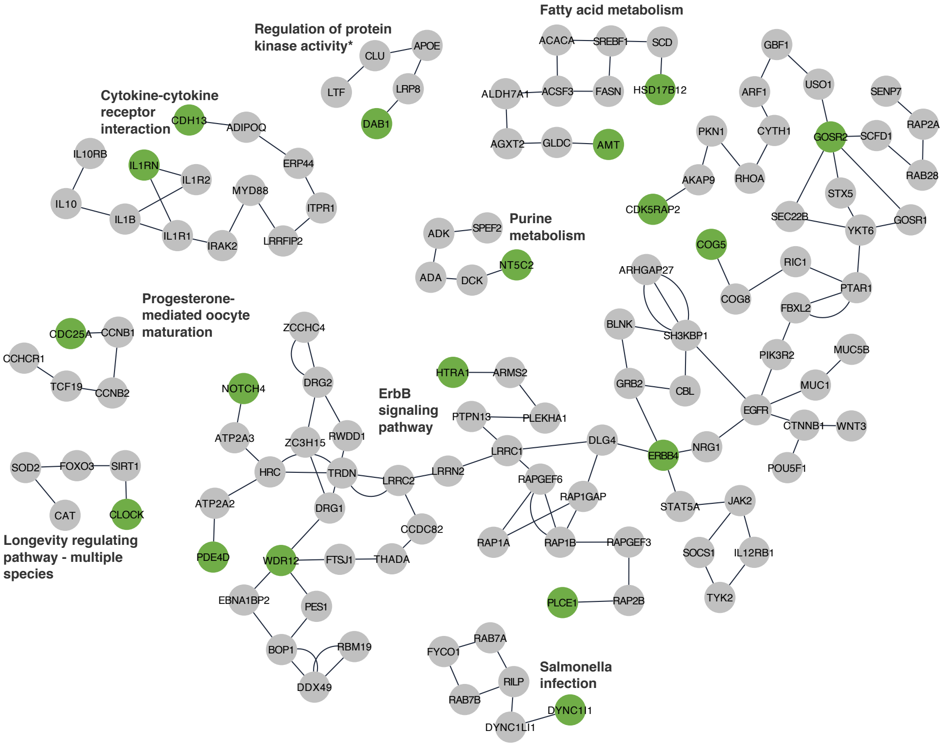

Supplementary Figure 15 | Protein interaction network for brain tissue coronary artery disease association in the hospitalised phenotype. The STRING protein interaction network shows the hospitalised CAD-associated gene products (i.e. proteins at Level 4; Figure 3b) and their interacting partners from Levels 0 to 3. The association is driven by brain tissue-specific eQTLs derived from the brain gene regulatory network. The protein network visualises the CAD-association as an entire protein network, drawing out the data, highlighting the interacting partners and their pathways. The level 4 proteins (the encoding genes and their eQTLs) enriched for CAD, are coloured green (n = 18). Grey coloured proteins (n = 124) are interacting partners forming the network from level 0 to level 3. There are a total of 18 CAD-genes and 30 CAD-eQTLs (Supplementary Table 10a) driving this association. The top KEGG pathway or *gene ontology: biological process (Supplementary Table 10b) is listed next to each protein cluster. The genes (e.g. *ERBB4, NOTCH4, HSD17B12)* and pathways (e.g. "ErbB signaling pathway" and "fatty acid metabolism") have relevance to CAD and SARS-CoV-2. Cytoscape (version 3.8.2) was used for visualising the network.

Extended Data – Supplementary Tables

Supplementary Table 1:	Summary of GWAS input data from COVID-19 HGI
Supplementary Table 2:	CoDeS3D results assessing regulatory effects of severe and hospitalised SNPs in the lung
Supplementary Table 3:	Protein interaction network details (SNP, gene, and trait associations derived from STRING) for all tissues (blood, brain, lung, and coronary artery) across severe and hospitalised phenotypes prior to bootstrapping
Supplementary Table 4:	Protein interaction network enrichments (derived from STRING and PROPER-Seq) for hospitalised and severe phenotypes in lung tissue
Supplementary Table 5:	Druggable genome results for the hospitalised lung protein interaction network genes at all levels
Supplementary Table 6:	CoDeS3D results assessing regulatory effects of severe and hospitalised SNPs in blood, brain, and coronary artery tissues
Supplementary Table 7:	Summary statistics of the gene regulatory networks in brain, lung, and the coronary artery
Supplementary Table 8:	Protein interaction network enrichments (derived from STRING and PROPER-Seq) for hospitalised and severe phenotypes in blood, brain, and coronary artery tissues
Supplementary Table 9:	Coronary artery disease comorbidity in brain tissue - protein interaction network
Supplementary Table 10:	Cardiovascular disease comorbidity in lung tissue – protein interaction network
Supplementary Table 11:	The code and data sources used in the analysis
